# The Efficacy of Alternative, Environmentally Friendly Plant Protection Measures for Control of Fall Armyworm, *Spodoptera Frugiperda*, in Maize

**DOI:** 10.3390/insects11040240

**Published:** 2020-04-10

**Authors:** Dirk Babendreier, Lakpo Koku Agboyi, Patrick Beseh, Michael Osae, Jerry Nboyine, Selorm E. K. Ofori, Justice O. Frimpong, Victor Attuquaye Clottey, Marc Kenis

**Affiliations:** 1 CABI Europe-Switzerland, Rue des Grillons 1, CH-2800 Delémont, Switzerland; m.kenis@cabi.org; 2 CABI, P.O. Box CT 8630, Cantonments, Accra GA 0376800, Ghana; l.agboyi@cabi.org (L.K.A.); v.clottey@cabi.org (V.A.C.); 3 Plant Protection and Regulatory Services Directorate (PPRSD), P.O. Box M37, Accra 00495426, Ghana; pkbeseh@gmail.com; 4 Radiation Entomology and Pest Management Centre, Biotechnology and Nuclear Agriculture Research Institute, Ghana Atomic Energy Commission, P.O. Box LG80, Legon, Accra, Ghana; mosae5@yahoo.com (M.O.); selormofori@yahoo.com (S.E.K.O.); 5 Savanna Agricultural Research Institute, Council for Scientific and Industrial Research, P.O. Box 52, Tamale NL-1252, Ghana; nboyinejerry@yahoo.co.uk; 6 Soil and Environmental Sciences Research Centre, Biotechnology and Nuclear Agriculture Research Institute, Ghana Atomic Energy Commission, P.O. Box LG80, Legon, Accra, Ghana; justicefrimpong85@gmail.com

**Keywords:** sustainable pest management, fall armyworm, biopesticides, maize, Ghana, field trials

## Abstract

The invasive fall armyworm (FAW) is threatening maize production and the livelihoods of millions of smallholder farmers in the newly invaded areas in Africa and Asia. To control this new key pest and to overcome health, environmental, and resistance problems related to the indiscriminate use of insecticides, effective and sustainable alternative pest control approaches are needed. Here, we report on field trials that tested maltodextrin, neem-based products, ash, and soil, as well as the locally produced alata samina soap, in the Upper West and Greater Accra regions, Ghana. Significant reductions of larval numbers and crop damage, together with increased yields, were mostly achieved by applying the insecticide emamectin benzoate, which was considered the positive control in this set of trials. However, high efficiency and cost–benefit ratios were also achieved with two neem-based products. Maltodextrin was only efficient at one of the two sites, with a clear dose-dependent effect, while the higher dosage was nearly as effective as emamectin benzoate. Due to its relatively high product cost, maltodextrin is generally less cost-efficient. Ash and soil, as well as alata samina soap treatments, did not efficiently reduce FAW larval numbers or crop damage at the dosages tested; thus, they also did not significantly increase maize yields.

## 1. Introduction

The fall armyworm, *Spodoptera frugiperda* (J.E. Smith), is a polyphagous migratory insect pest that is able to cause considerable economic losses in over 80 different crops [1]. The pest is native to the tropical and sub-tropical regions of North, Central, and South America, where it has been considered a key pest in maize and several other crops for decades. Fall armyworm was detected for the first time on the African continent in January 2016 in Nigeria [2], and by 2019 had been reported in almost all of sub-Saharan Africa, as well as in South- and Southeast Asia, causing substantial yield losses [3,4]. Recently, published pest distribution and climatic suitability models have indicated that the environmental requirements for this pest to establish itself permanently and thrive are present through large parts of Africa, the warmer regions of Asia, and even some parts of southern Europe [5].

As maize is a staple food in most African countries and also the second most important crop in Asia, the invasion of *S. frugiperda* threatens the food security of hundreds of million people. Farmers in the invaded area were not prepared for this devastating pest, resulting in heavy losses on one hand, but also a drastic increase of insecticide use on the other [6]. Farm households are reported to often use highly hazardous or banned products with generally no appropriate personal protective equipment [7]. Such over-reliance on insecticides is highly problematic due to potential environmental and health risks, as well as the strong capability of the fall armyworm to quickly develop resistance (e.g., Zhu et al. [8]). Moreover, numerous applications of insecticides are increasing production costs; in particular, the often-used broad-spectrum products may disrupt current production systems of small holder farmers, which are often based on low inputs and a certain reliance on natural biological control (e.g., Meagher et al. [9]). Therefore, this shift to applying pesticides is not in line with integrated pest management approaches and is unsustainable in the long run.

Aiming to solve these problem more sustainably, the importation of natural enemies from the native area of the pest for release and permanent establishment in invaded areas (classical biological control) may be considered. A candidate for biological control, *Telenomus remus*, is already present in Africa [10], so it remains to be seen whether this egg parasitoid can potentially be mass produced and released as an inundative biological control agent [11]. Currently, no commercial production is in place in Africa and the application of this approach might be challenging, considering the current financial power of smallholder farmers.

Altogether, a strong need exists to identify alternative solutions for farmers that would be effective, affordable, in line with Integrated Pest Management (IPM), and tailored to suite the local conditions and farming practices in Africa [12,13,14]. It is obvious to consider the actions smallholder farmers in Central America and Mexico are taking, as they have been managing fall armyworm (FAW) successfully for hundreds of years, even though the differences in agricultural systems should be accounted for. For instance, cultural techniques such as early planting have been suggested to contribute to successful FAW management [15]. Intercropping maize with legumes (e.g., beans) may also reduce FAW damage, especially when beans are sown earlier than maize [16]. Push–pull approaches have been shown to reduce populations of FAW, in some cases substantially, under African conditions [16,17], and a recent review also mentioned sound fertilization, soil management, and habitat diversification as factors able to reduce FAW incidence [13]. However, these methods might not be feasible for all small holder farmers in SSA. For example, for the push–pull system specifically, farmers might lack opportunities to feed Napier grass to livestock, or generally the suggested methods might not have sufficient levels of control or might be too expensive (including opportunity costs). Therefore, as alternatives to chemical pesticides for direct control measures of FAW, biopesticides have been considered multiple times [12,18].

A recent review on the potential of microbial substances, including entomopathogenic fungi, entomopathogenic nematodes, bacteria, and baculoviruses, to control FAW was published by [19]. Despite a paucity of good field studies, there is evidence that some microbials may be very effective in controlling FAW. In particular, products based on Bt toxins and baculoviruses are already registered in some newly infested countries, and more products might enter the market soon. In Ghana specifically, the government has switched from supporting chemical pesticides to Bt-based products recently. Other biopesticides are based on pods or leaves from neem trees (*Azadirachta indica* Juss.), of which extracts from pods or leaves are known to possess pesticidal properties [20,21,22], and which are readily available across most of Africa. Another insecticidal product that is known to be effective against a range of insect pests is maltodextrin. This is a physically acting organic insecticide made from starch, vegetable oil, and water, which is relatively fast-acting and causes death by suffocation through blocking the spiracles [23]. Soap has also been used effectively to manage insect pests, particularly for soft-bodied insects such as aphids, some scales, psyllids, whiteflies, mealybugs, thrips, and spider mites [24]. Even though the mode-of-action of insecticidal soaps is not clearly understood, it is known that target pests need to make contact with the soap solution. Anecdotal reports from farmers indicate that soap sprayed into the whorl may also be used to control FAW in maize. Apart from soap, farmers also claim that placing soil or wood ash in the whorl of maize reduces FAW infestation and damage. Ash may block spiracles of the target insect, leading to suffocation and death, while soil may prevent access to the plant by the pest or also affect the larvae through abrasion. Soil and ash have been used for control of FAW for a long time by small holder farmers in the Americas [25], and generally also in Africa [26,27].

In general, all of the above alternative solutions barely have a pre-harvest interval, have no relevant residues, and are safe for applicators and consumers. However, rigorous testing of their efficacy in the context of African small holder farmers is mostly lacking. Hence, field trials were conducted in Ghana, with the aim of testing the efficacy of the aforementioned indigenous strategies for the management of FAW in maize, and ultimately with the aim of developing recommendations that can be used at scale.

## 2. Materials and Methods

### 2.1. Study Sites

This field experiment was established at two study sites. The first site (site 1) was located at the Council for Scientific and Industrial Research—Savanna Agricultural Research Institute (CSIR-SARI) at Tanina in the Wa West District of the Upper West region, Ghana. The site is located at an altitude of 368 m and belongs to the Guinea Savanna zone. The region has a unimodal rainfall pattern with a peak between May and October, and a mean total annual rainfall of about 1050 mm. During the main 12 weeks of trial implementation, 405 mm of rain were observed in total, with a weekly mean of 33.7 ± 17.5 mm. Planting was done on 18th July 2018 after the first rains provided good conditions. Approximately 470 mm of rain was subsequently observed between mid-July and the end of October, when the trial crop was harvested. Basal fertilizer application of 100 kg/ha NPK (23-10-5) was done two weeks after crop emergence, while top dressing with 50 kg/ha sulphate of ammonia (21% N) was done three weeks after basal application. Weed control was done manually. The soil in the experimental area belongs to the Savannah Ochrosol type, with a relatively thin layer of top soil (about 25 cm deep) consisting of greyish brown loamy sand.

The second site (site 2) was located at the research farm of the Biotechnology and Nuclear Agriculture Research Institute (BNARI) of the Ghana Atomic Energy Commission, located in the Ga East Municipality of the Greater Accra Region of Ghana, at 52 m above sea level, with rainfall following a bimodal pattern. The experiment was carried out during the minor cropping season, which shows rather erratic precipitation. During the main 12 weeks of trial implementation, 1383 mm of rain was observed in total, which is more than the long-term average, with a weekly mean of 115.2 ± 84.3 mm. Planting was done on 6th September. The soils of the study area belong to the Savannah Ochrosols subgroup, which is characterized by very shallow, reddish-brown and brown concretionary soil, which is medium to light textured. For weed control, a pre-emergence herbicide was sprayed the day after sowing (Lumax^®^, 537.5SE), with manual weeding afterwards as needed. Two weeks after crop emergence, 100 kg/ha NPK (15-15-15) was applied to maize seedlings, with about 2 g placed near individual plants into small holes. A month after the initial fertilizer application, 50 kg/ha of sulphate of ammonia was applied to the crop.

At both sites, the fields were tractor ploughed followed by harrowing. Maize has been one of the major crops planted before the arrival of the fall armyworm, and FAW has been shown to be present in high numbers at both sites.

### 2.2. General Experimental Design

In total, four trials were conducted to test the efficiency of (1) maltodextrin, (2) neem products, (3) ash and soil, as well as (4) alata samina (a local soap). Unless otherwise specified, the following applies to all four trials, while for treatment details we like to refer to the specific trial description (in Section 2.3, Section 2.4, Section 2.5 and Section 2.6). For all trials, plots measuring 6.3 x 10 m, separated from each other by 2 m alleys, were planted with the open pollinated seed maize variety Obatanpa, with inter- and intrarow spacings of 70 and 30 cm, respectively, with two seeds per hill. Two weeks after emergence, they were thinned to a single plant per hill. All four trials were set up following a randomized complete block design with 4 replicates at each of the two sites.

Monitoring of the complete trial site for FAW was carried out regularly immediately after emergence of the crop. The treatments were imposed when approximately 10% of plants overall were showing signs of infestations with FAW. The first application of treatments was done on 15th August at site 1 and on 19th September, 2018, at site 2. Two more applications were then done at site 1, while only one more application was done at site 2. The treatments were applied using knapsack sprayers, which were always thoroughly cleaned after use. Treatments were done until run-off from the plants started to occur, based on approximately 200 l/ha early in the cropping stage and 300 l/ha after 36 days of crop establishment.

During the trial period, data were generally collected directly before treatments were applied and seven days afterwards, resulting in six assessment dates for site 1 and four for site 2. On each assessment date, 20 plants per plot were sampled by walking 2 times diagonally through the plot, assessing 10 plants each. The following data were collected per plot by summing up the figures from the 20 plants: the number of egg masses; the number of larvae (non-destructively); crop damage, for which the Davis scale (from 0—no damage, to 9—heavy damage) was used to assess damage by FAW to maize plants; phytotoxicity, including modifications in color, necrosis, or deformations on sampled plants; and key natural enemies (i.e., the number of spiders, ladybird beetles, and earwigs).

In addition, during harvest, the percentage of cobs with characteristic signs of FAW damage was assessed based on 20 randomly selected plants per plot (site 1) or 40 randomly chosen cobs (site 2). Finally, the yield was assessed per plot by collecting all healthy grains from the 6 inner rows (neglecting the outer two from the total of 8 rows per plot) and weighing these after the grains had been sun-dried to 12% moisture content.

### 2.3. Maltodextrin

In this trial, three maltodextrin treatments (0.27%, 0.53% (the recommended dose), and 0.8%) were established, based on Eradicoat containing maltodextrin (282 g/l), obtained from Certis UK and Ireland. In addition, a negative control (no insecticide) and a positive control (Ema 19.2 EC, emamectin benzoate, Adama West Africa, Ltd.) at 0.17% were also implemented. Emamectin benzoate is an insecticide based on emamectin, which is produced from fermentation of the soil actinomycete *Streptomyces avermitilis*. It is not known to cause human health hazards, but is toxic to honeybees and aquatic organisms [12]. Treatments were applied at three weeks intervals.

### 2.4. Neem

In total, the following six treatments were implemented for this neem trial: Grow-Safe neem oil, applied at 0.17% (low dose); Grow-Safe neem oil, applied at 0.33% (normal dose); Ozoneem oil, applied at 0.17% (low dose); Ozoneem oil, applied at 0.33% (normal dose); the untreated control; and emamectin benzoate at 0.17% as a positive control. Grow-Safe neem oil is produced by Green-Gro Ltd., Ghana, and was purchased from the manufacturer, while Ozoneem oil, produced by Ozone Biotech, India, was purchased from Karsam Macro Ltd., Ghana. Grow-Safe was mixed with liquid soap (provided by the manufacturer) at a concentration of 0.67 ml/l solution before application, but Ozoneem oil was applied without extra additives because this commercial product already comprises the complete formulation. Treatments were applied at three weeks intervals.

### 2.5. Soil and Ash

Soil material was collected from the experimental fields (topmost part of the experimental field) and ash was collected from local firewood from a designated team member’s house. In addition to the positive (emamectin benzoate at 0.17%) and negative controls, the following four treatments were applied: (1) Ash applied by hand, 2–3 ml per whorl, equaling about 650 ml of ash per plot. As the crop matured (from 36 days after sowing onwards), the dosage was doubled to 5 ml per whorl. (2) Ash solution applied by knapsack sprayer—650 ml of ash was mixed with 2.25 l of water (twice the amount of water than normally used for such area). As the crop matured (from 36 days after sowing onwards), the dosage was doubled to 1300 ml of ash per plot, mixed with 4.5 l of water per plot. (3) Local soil, sieved with a 2 mm mesh and applied by hand. For this, 2–3 ml per whorl were applied during early crop stages and the amount was doubled as the crop matured. (4) Local soil mixed with ash from local firewood, at a ratio of 50:50 and at 2–3 ml per whorl, which was again doubled as the crop matured. Unlike for the neem and maltodextrin trials, treatments here were applied at biweekly intervals at site 1.

### 2.6. Alata Samina

The alata samina soap was purchased from vendors in the markets of Wa and Accra. This soap is made from the ash of the barks of plants that are locally harvested, such as plantain, palm tree leaves, and shea tree bark. It was reported to be effective against insect pests by reducing their population densities in eggplant and okra farms in Ghana [27]. Prior to being sprayed on maize plants, alata samina was dissolved in water and the following four weight/volume concentrations of alata samina were tested: 0.033%, 0.066%, 0.1%, and 0.133%. In addition, a positive control based on emamectin benzoate (0.167%) and one untreated control were implemented. Alata samina was applied at biweekly intervals at site 1, in line with the supplier’s recommendation in this area.

### 2.7. Data Analysis

For each trial, data were analyzed separately by generalized linear models, followed by Least Significant Difference post hoc tests and focusing on between treatment differences for the following factors: egg mass numbers, larval numbers, plant damage, cob damage, and yield. The phytotoxicity and number of natural enemies were not statistically analyzed because of very low overall numbers (except for the maltodextrin trial). All analyses were performed with SPSS 25.0.

Partial budget analysis was used to assess the cost–benefit ratio of all treatments implemented in the study. This was calculated from grain yields (at the market price for maize in either Northern Ghana (GH¢ 1.4/Kg) or Southern Ghana (GH¢ 2/Kg) from early 2019) of individual treatments and the cost of the applied insecticides, biopesticides, or natural products compared to the untreated control. The following costs were calculated for the applied products, based on one treatment and one ha: emamectin benzoate, GH¢ 20; maltodextrin, GH¢ 156 (high dose); Ozoneem, GH¢ 90 (high dose); Grow-Safe, GH¢ 38 (high dose); alata samina (local soap), GH¢ 1.33 (high dose). Labor costs for spraying were assumed to be GH¢40/ha in Northern Ghana and GH¢50/ha in Southern Ghana. The same amount was assumed as for the application of sand or ash treatments.

The net profit was calculated as the additional yield harvested/ha (in kg) as compared to the untreated control x the market price – total costs of treatments (the sum of product and labor costs). The cost/benefit ratio was then calculated by dividing the net profit by the total costs of respective treatments, indicating how many additional GH¢ one would get per 1 GH¢ invested in plant protection.

## 3. Results

### 3.1. Maltodextrin

The number of FAW egg masses was assessed throughout the various trials, but was only analyzed statistically for the first assessment date at site 2, where between 5 and 7.5 egg masses per 20 plants were found, with no significant differences across treatments (χ^2^_4,15_ = 5.6, *p* = 0.23). During later assessment dates at site 2 and altogether at site 1, the number of egg masses found was too low (i.e., mostly zero) to allow for statistical analysis. Similarly, the number of natural enemies was too low at site 1 to allow for statistical analysis. At site 2, numbers were generally also low (between 1 and 5 per 20 plants on average across all dates and treatments), with no significant differences during the first (χ^2^_4,15_ = 4.2, *p* = 0.38), second (χ^2^_4,15_ = 7.7, *p* = 0.10), third (χ^2^_4,15_ = 8.1, *p* = 0.09), and fourth assessment dates (χ^2^_4,15_ = 0.95, *p* = 0.92).

**Figure 1:**
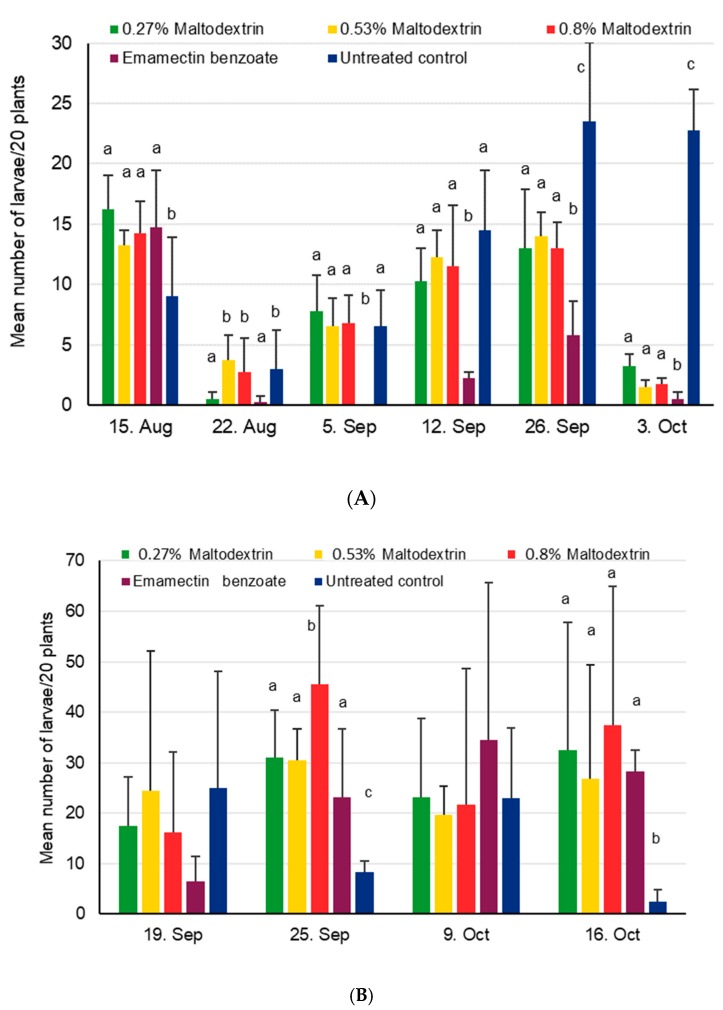
Mean number of fall armyworm (FAW) larvae + SD found on 20 maize plants in the maltodextrin trial during vegetative growing stages conducted in 2018. Within a given date, bars sharing the same letters are not significantly different from each other. If no letters are shown this indicates that no differences were found. (**A**) Site 1 near Wa, Upper West, Ghana, where treatments were applied on 16 August, 6 September, and 27 September. (**B**) Site 2 near Accra, Ghana, where treatments were applied on 20 September and 10 October.

FAW larval numbers were significantly different among treatments on 5 August (χ^2^_4,15_ = 12.8, *p* = 0.012), 12 August (χ^2^_4,15_ = 11.7, *p* = 0.019), 5 September (χ^2^_4,15_ = 35.5, *p* < 0.001), 12 September (χ^2^_4,15_ = 21.2, *p* < 0.001), 26 September (χ^2^_4,15_ = 50.9, *p* < 0.001), and 3 October (χ^2^_4,15_ = 708, *p* < 0.001), and went down after the first and third application dates at site 1 but not after the second (see Figure 1). Except for the pre-treatment assessment, the lowest numbers of larvae were consistently observed for the positive control, becoming significantly different from all other treatments from the third assessment date onwards (*p* < 0.05, Figure 1). The maltodextrin treatments were significantly different from the negative control only at the last two assessment dates and were most pronounced at the last date, but did not show a clear dose-dependent effect (Figure 1). At site 2 in contrast, no clear pattern could be seen, which was linked to very high data variability. Lowest larval numbers were even observed in the untreated control for most of the trial, resulting in significant treatment effects on 25 September (χ^2^_4,15_ = 20.2, *p* < 0.001) and 16 October (χ^2^_4,15_ = 10.1, *p* = 0.038). No significant treatment effects were observed for the first assessment that was done before treatments were applied (χ^2^_4,15_ = 3.6, *p* = 0.46). or for the assessment on the 9th October, which occurred shortly before the second application date (χ^2^_4,15_ = 1.7, *p* = 0.80). In general, higher numbers of larvae were observed at site 2 compared to site 1, with regularly more than 1 larvae found per plant.

**Figure 2:**
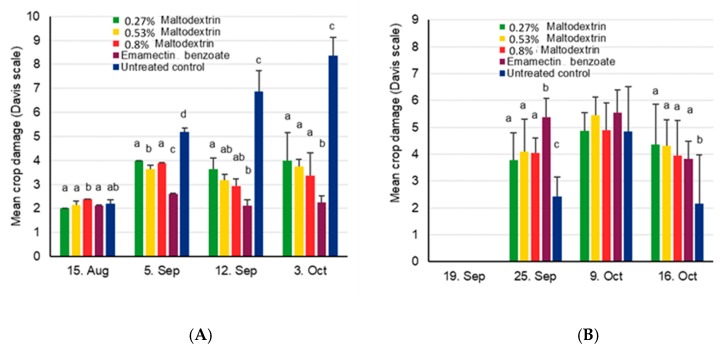
Mean crop damage induced by FAW + SD found in the maltodextrin trial during vegetative growing stages conducted in 2018. Within a given date, bars sharing the same letters are not significantly different from each other. If no letters are shown, this indicates that no differences were found. (**A**) Site 1 near Wa, Upper West, Ghana, where treatments were applied on 16 August, 6 September, and 27 September. (**B**) Site 2 near Accra, Ghana, where treatments were applied on 20 September and 10 October.

Damage scores were similar at site 1 in the beginning of the experiment at a level of around 2, but increased to a high level of 8.4 (according to Davis scale) for the untreated control towards the end of the vegetative growth period (Figure 2); in the other treatments, scores were between 2 and 4, with the lowest numbers observed in the positive control and slightly higher values for maltodextrin. Significant differences were found for assessments done on 15 August (χ^2^_4,15_ = 36.7, *p* < 0.001), 5 September (χ^2^_4,15_ = 1518, *p* < 0.001), 12 September (χ^2^_4,15_ = 307, *p* < 0.001), and 3 October (χ^2^_4,15_ = 266, *p* < 0.001) (Figure 2). At site 2, crop damage was zero during the first assessment on 19th September, quickly rising to levels of 4 or more for all treatments except the untreated control. Significant differences were found for assessments done on 25 September (χ^2^_4,15_ = 30.8, *p* < 0.001) and 16 October (χ^2^_4,15_ = 10.1, *p* = 0.038), with the positive control showing higher damage levels than the untreated control, but not for 9 October (χ^2^_4,15_ = 2.4, *p* = 0.67; Figure 2) (i.e., before the second application date).

**Figure 3:**
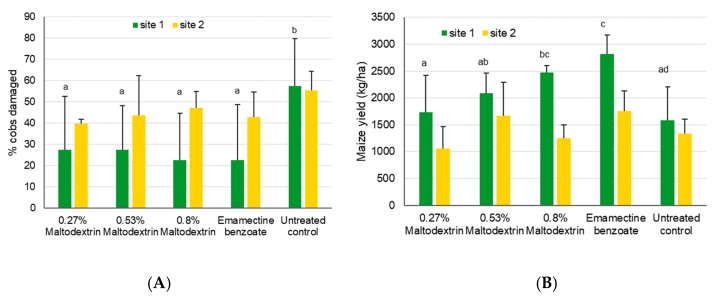
Mean maize cob damage induced by FAW + SD (**A**) and mean maize yields (**B**) after treatment with various concentrations of maltodextrin for site 1 near Wa, Upper West, Ghana, and site 2 near Accra, Ghana. Bars for yield at site 1 sharing the same letters are not significantly different from each other for the yield data, while missing letters indicate that no significant differences were found.

Cob damage was very highly variable, but significant differences were found for site 1, where cob damage was more than twice as high for the untreated control compared to other treatments (χ^2^_4,15_ = 12.9, *p* = 0.012) (Figure 3). No significant differences were found for cob damage at site 2 (χ^2^_4,15_ = 6.0, *p* = 0.20). The yield at site 1 was between 1.5 and 2.8 tons/ha, with significant differences between treatments (χ^2^_4,15_ = 24.6, *p* < 0.001), while the untreated control showing significantly less yield than the positive control and the high concentration maltodextrin treatment (*p* < 0.05). At site 2, the yield varied between 1.0 and 1.7 tons/ha, without showing significant treatment effects (χ^2^_4,15_ = 8.4, *p* = 0.063).

The yield increase at site 1 resulted in positive returns for the maltodextrin treatments, but also due to relatively high product costs, these were lower as compared to the positive control (Table 1). Less promising were the findings at site 2, where in addition to the positive control, a slightly positive return was only found for the 0.53% concentration maltodextrin treatment.

### 3.2. Neem

**Figure 4:**
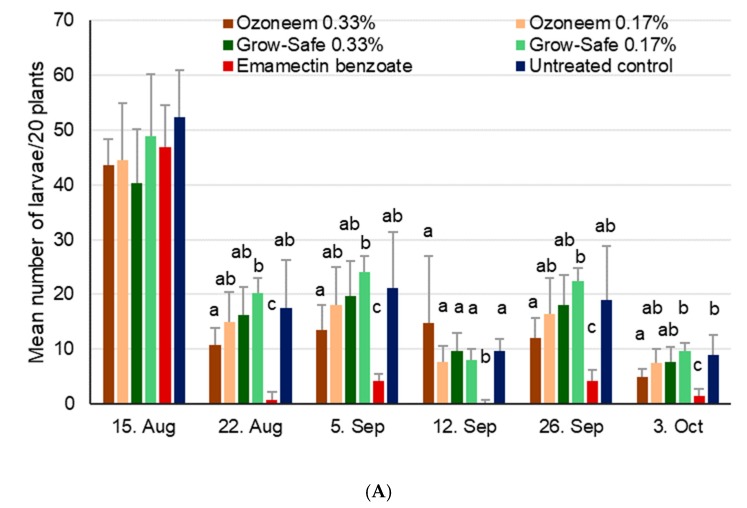

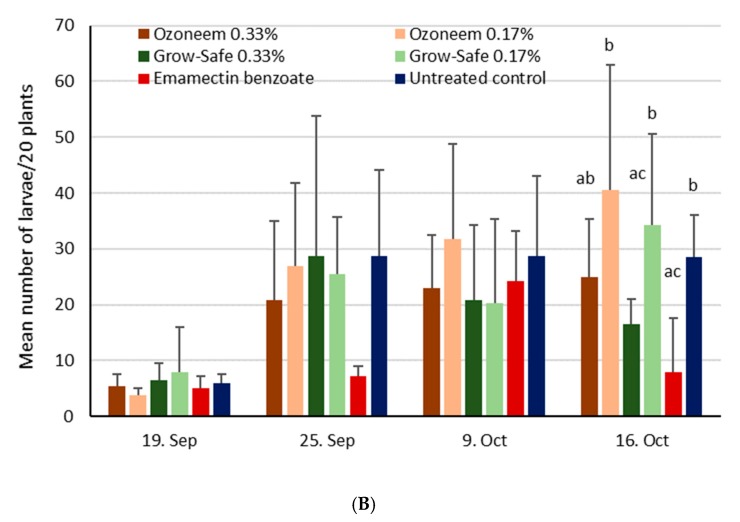
Mean number of FAW larvae + SD found on 20 maize plants in the neem trial during vegetative growing stages conducted in 2018. Within a given date, bars sharing the same letters are not significantly different from each other. (**A**) Site 1 near Wa, Upper West, Ghana, where treatments were applied on 16 August, 6 September, and 27 September. (**B**) Site 2 near Accra, Ghana, where treatments were applied on 20 September and 10 October.

The number of FAW egg masses was not statistically different for the first (χ^2^_5,18_ = 5.8, *p* = 0.32), second (χ^2^_5,18_ = 5.1, *p* = 0.40), or third assessment dates at site 2 (χ^2^_5,18_ = 1.5, *p* = 0.91), with insufficient numbers for analysis during later dates, as well as for site 1. Larval numbers at site 1 were not significantly different among treatments during the first assessment date (χ^2^_5,18_ = 5.8, *p* = 0.33), but were different after first application of various products, for example on the 22nd August (χ^2^_5,18_ = 50.8, *p* < 0.001), as well as the following dates (5 Sep, χ^2^_5,18_ = 35.7, *p* < 0.001; 12 Sep, χ^2^_5,18_ = 19.8, *p* = 0.001; 26 Sep, χ^2^_5,18_ = 34.0, *p* < 0.001; 3 Oct, χ^2^_5,18_ = 44.6, *p* < 0.001). Generally, numbers of larvae/20 plants went down over time for all treatments, while the positive control always had the lowest number of larvae and the neem treatments overall showed similar larval numbers compared to the untreated control (Figure 4A).

At site 2, larval numbers were not different during the first (χ^2^_5,18_ = 3.8, *p* = 0.58), second (χ^2^_5,18_ = 7.8, *p* = 0.17), or third assessments (χ^2^_5,18_ = 3.1, *p* = 0.68), while significant differences were only found during the last assessment date on 16 October (χ^2^_5,18_ = 21.4, *p* < 0.001). Here, the positive control showed significantly less larvae as compared to the untreated control and the two neem treatments with the lower concentrations (*p* < 0.05, Figure 4B).

**Figure 5:**
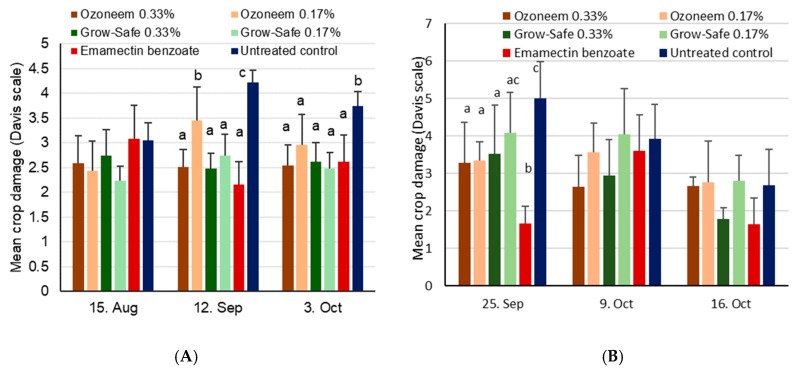
Mean crop damage induced by FAW + SD found in the neem trial during vegetative growing stages conducted in 2018. Within a given date, bars sharing the same letters are not significantly different from each other. If no letters are shown, this indicates that no differences were found. (**A**) Site 1 near Wa, Upper West, Ghana, where treatments were applied on 16 August, 6 September, and 27 September. (**B**) Site 2 near Accra, Ghana, where treatments were applied on 20 September and 10 October.

Maize damage scores at site 1 (according to the Davis scale) were not significantly different during the first assessment data period (i.e., before treatments were applied; χ^2^_5,18_ = 9.3, *p* = 0.10) (Figure 5). Four weeks later, on 12 September, and after treatments had been applied twice, significant differences were obtained (χ^2^_5,18_ = 82.8, *p* < 0.001), with higher values shown for the Ozoneem 0.17% treatment and the untreated control. Significant differences in maize plant damage were also found at the last assessment date (χ^2^_5,18_ = 30.7, *p* < 0.001), with all treatments showing similar values of 2.5 to 3, except the untreated control. At site 2, virtually no damage was found on plants before treatments were applied, but substantial damage was observed on 25 September, with significant differences observed among treatments (χ^2^_5,18_ = 35.6, *p* < 0.001). Two more assessments of crop damage revealed no significant differences among treatments on 9 October (χ^2^_5,18_ = 9.3, *p* = 0.10) and 16 October (χ^2^_5,18_ = 11.2, *p* = 0.06).

**Figure 6:**
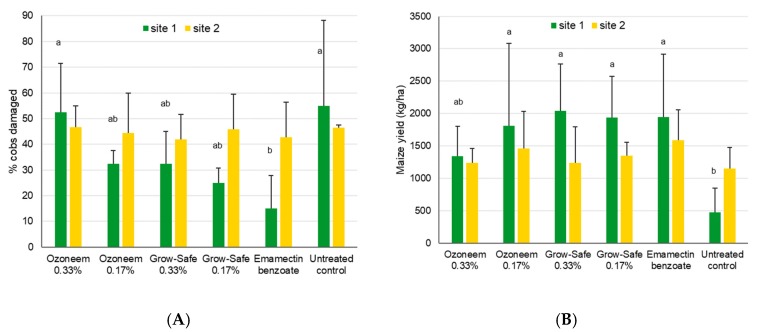
Mean maize cob damage induced by FAW + SD (**A**) and mean maize yields (**B**) after treatment with two neem-based products for site 1 near Wa, Upper West, Ghana, and site 2 near Accra, Ghana. Bars sharing the same letters are not significantly different from each other for the yield data, while missing letters indicate that no significant differences were found (i.e., for site 2).

Significant differences in cob damage were found at site 1 (χ^2^_5,18_ = 21.2, *p* = 0.001) with lower damage for the emamectin treatment when compared to the untreated control and the Ozoneem 0.33% treatment (*p*<0.05, Figure 6). Yield differences were also observed at site 1 (χ^2^_5,18_ = 15.1, *p* = 0.01), with lower yields for the untreated control compared to all treatments except for Ozoneem 0.33% (*p*<0.05). No significant differences among treatments were found at site 2, neither for cob damage (χ^2^_5,18_ = 0.84, *p* = 0.97) nor for the yield (χ^2^_5,18_ = 4.16, *p* = 0.53).

At site 1, very positive returns were obtained, in particular for the Grow-Safe product for which returns were in the same range as the positive control (Table 2). Less promising again were the findings at site 2, where positive returns were only found for the lower concentrations of the two neem products.

### 3.3. Ash and Soil

**Figure 7:**
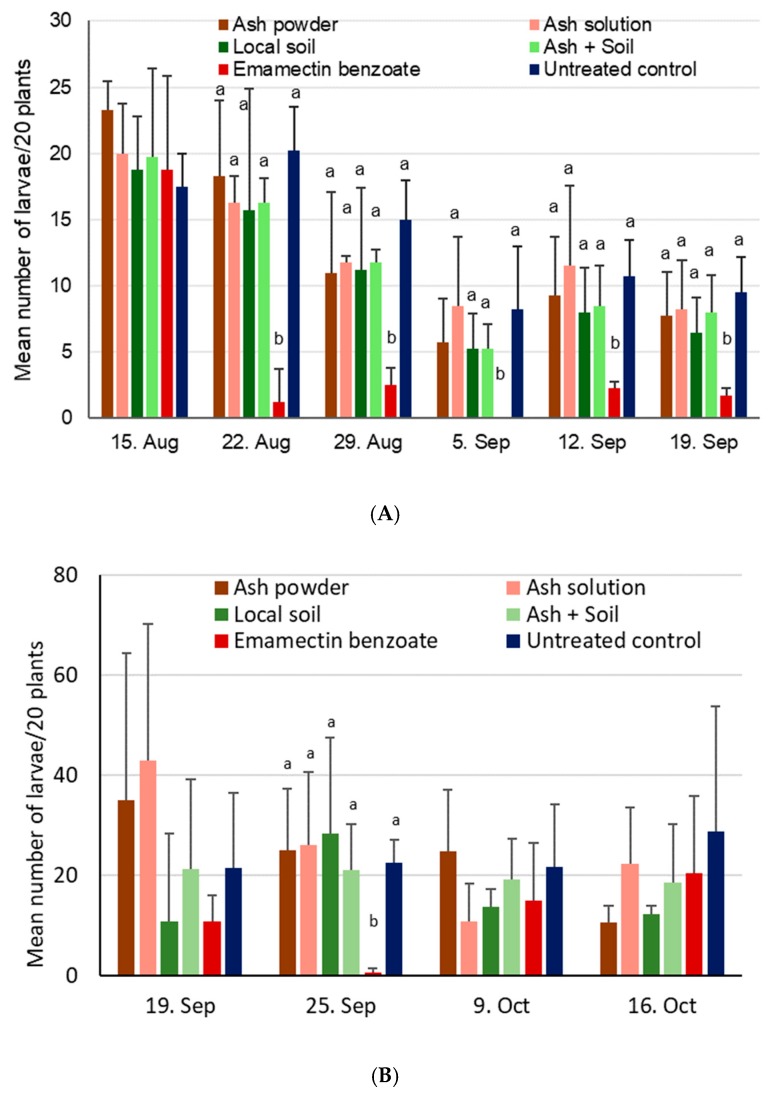
Mean number of FAW larvae + SD found on 20 maize plants after application of ash- or soil-based treatments during vegetative growing stages conducted in the 2018 season. Within a given date, treatment bars sharing the same letters are not significantly different from each other. (**A**) Site 1 near Wa, Upper West, Ghana, where treatments were applied on 16 August, 30 August, and 13 September, (**B**) Site 2 near Accra, Ghana, where treatments were applied on 20 September and 10 October.

The number of FAW egg masses was not statistically analyzed because virtually no egg masses were found altogether (again, in particular in site 1). Those egg masses found at site 2 were mostly from the positive control. Larval numbers at site 1 were not significantly different among treatments during the first assessment date (χ^2^_5,18_ = 4.5, *p* = 0.48), but were different after the first application of the various products (i.e., the 22 August, χ^2^_5,18_ = 52.1, *p* < 0.001), as well as the following dates (i.e., 29 Aug, χ^2^_5,18_ = 32.7, *p* < 0.001; 5 Sep, χ^2^_5,18_ = 21.3, *p* = 0.001; 12 Sep, χ^2^_5,18_ = 20.5, *p* = 0.001; 19 Sep, χ^2^_5,18_ = 25.5, *p* < 0.001. Overall, the numbers of larvae/20 plants went down over time for all treatments, including the untreated control. While the positive control always had the lowest number of larvae, which was significantly different from all other treatments, all other treatments did not show any significant differences among themselves (Figure 7A).

At site 2, larval numbers were not different during the first assessment on 19 September (χ^2^_5,18_ = 10.2, *p* = 0.07), but became significantly different at the second date on 25 September (i.e., after application of the treatments; χ^2^_5,18_ = 19.4, *p* = 0.002), basically due to near-zero values for the insecticide control, with other treatments showing relatively little variation with between 21 and 28 larvae/20 plants. No differences were again found on the third (χ^2^_5,18_ = 7.8, *p* = 0.17) or the last assessment date on 3 October (i.e., after the second application of treatments; χ^2^_5,18_ = 6.4, *p* = 0.27) (Figure 7B).

**Figure 8:**
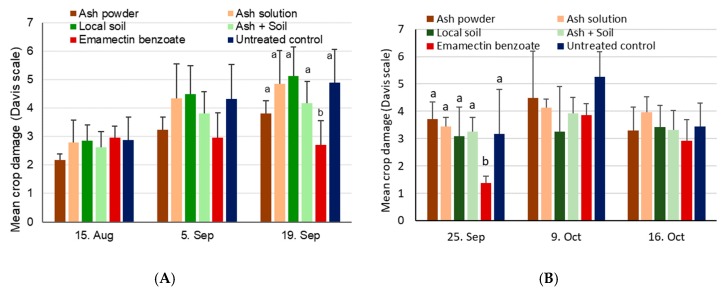
Mean crop damage induced by FAW + SD after application of ash- or soil-based treatments during vegetative growing stages conducted in the 2018 season. Within a given date, bars sharing the same letters are not significantly different from each other, while no letter indicates a lack of difference. (**A**) Site 1 near Wa, Upper West, Ghana, where treatments were applied on 16 August, 30 August, and 13 September. (**B**) Site 2 near Accra, Ghana, where treatments were applied on 20 September and 10 October.

Maize damage scores at site 1 were not significantly different during the first assessment date on 15 August before treatments were applied (χ^2^_5,18_ = 6.3, *p* = 0.28) (Figure 8A). Three weeks later on 5 September and after treatments had been applied twice, damage scores increased to 3–4.5, but were still without significant treatment differences (χ^2^_5,18_ = 10.6, *p* = 0.06). Significant differences in maize plant damage were, however, found during the last assessment date on 19 September (χ^2^_5,18_ = 25.0, *p* < 0.001), with the positive control showing significantly less damage compared to all other treatments. At site 2, virtually no damage was found on plants on 19 September before treatments were applied, but substantial damage was observed on 25 September, with significant differences among treatments (χ^2^_5,18_ = 24.0, *p* < 0.001) (Figure 8B). For the two subsequent assessments of crop damage, no significant differences were found among treatments on either 9 October (χ^2^_5,18_ = 10.4, *p* = 0.07) or 16 October (χ^2^_5,18_ = 5.4, *p* = 0.37).

**Figure 9:**
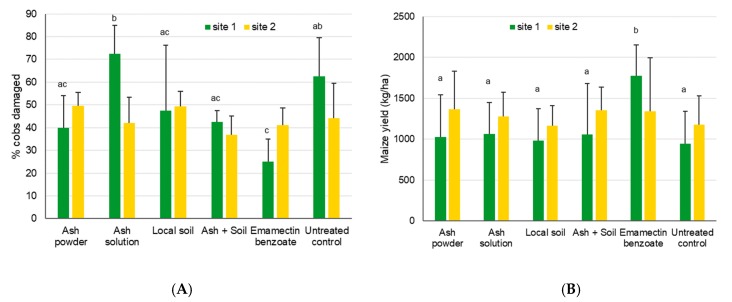
Mean maize cob damage induced by FAW + SD (**A**) and mean maize yields (**B**) after treatment with ash- or soil-based products for site 1 near Wa, Upper West, Ghana, and site 2 near Accra, Ghana. Bars sharing the same letters are not significantly different from each other, while missing letters indicate that no significant differences were found (i.e., for site 2).

Significant differences were found for cob damage at site 1 (χ^2^_5,18_ = 28.7, *p* < 0.001), with higher damage shown for the ash solution treatment and the untreated control when compared to the insecticide control (*p* < 0.05, Figure 9). Cob damage varied between 37% and 50% at site 2, with no significant differences observed among treatments (χ^2^_5,18_ = 6.9, *p* = 0.23).

Significant differences were found for the yield at site 1 only (χ^2^_5,18_ = 12.4, *p* = 0.03). While no differences could be found between the treatments involving ash and soil and the untreated control, a significantly higher yield was obtained for the positive control based on emamectin benzoate (see Figure 9). At site 2, the yield varied between 1.1 and 1.4 tons per ha, without showing significant treatment effects (χ^2^_5,18_ = 1.3, *p* = 0.94).

Despite no material costs being assumed for the ash and soil treatments, the corresponding cost–benefit figures were very small and even negative in some cases at both sites (Table 3). However, at least for site 2, the positive control also only showed a small positive value.

### 3.4. Alata Samina

**Figure 10:**
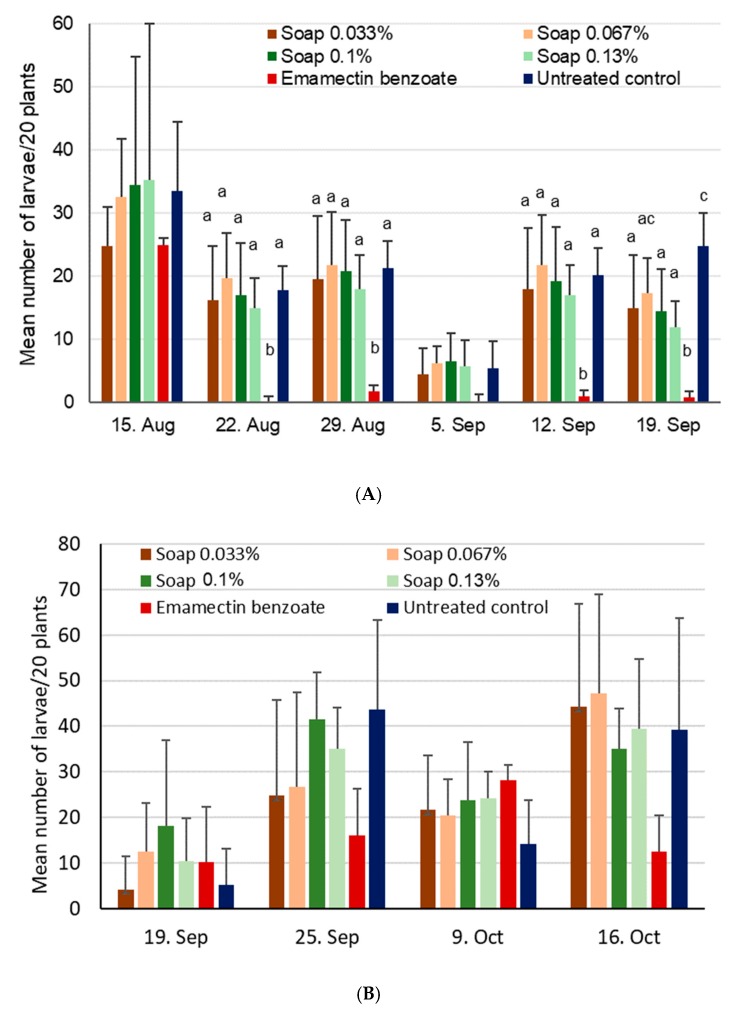
Mean number of FAW larvae + SD found on 20 maize plants after application of alata samina, a local soap, during vegetative growing stages conducted in the 2018 season. For a given date, treatment bars sharing the same letters are not significantly different from each other. (**A**) Site 1 near Wa, Upper West, Ghana, where treatments were applied on 16 August, 30 August, and 13 September. (**B**) Site 2 near Accra, Ghana, where treatments were applied on 20 September and 10 October.

Looking again at site 1 first, the numbers of FAW larvae were not statistically different for the first assessment date (χ^2^_5,18_ = 1.9, *p* = 0.96), but were different during the second date (22 Aug, χ^2^_5,18_ = 35.8, *p* < 0.001) and most of the following dates (29 Aug, χ^2^_5,18_ = 33.1, *p* < 0.001; 12 Sep, χ^2^_5,18_ = 34.4, *p* < 0.001; 19 Sep, χ^2^_5,18_ = 52.5, *p* < 0.001). Except for the assessment on 5 September (χ^2^_5,18_ = 11.0, *p* = 0.05), numbers of larvae stayed a relatively high level for all treatments, with only the positive control consistently showing low numbers (Figure 10A). It was only at the very last assessment date on 19 September that the soap treatments showed significantly lower numbers of larvae compared to the untreated control (*p* > 0.05).

At site 2, larval numbers were not significantly different during the first assessment (χ^2^_5,18_ = 5.1, *p* = 0.40) or during the second assessment, after applying initial (25 Sep, χ^2^_5,18_ = 4.6, *p* = 0.46) or subsequent treatments (9 Oct, χ^2^_5,18_ = 6.9, *p* = 0.23; 16 Oct, χ^2^_5,18_ = 8.0, *p* = 0.16). This was despite the positive control showing a rather low number of larvae and must be seen in the context of high observed variability (Figure 10B).

**Figure 11:**
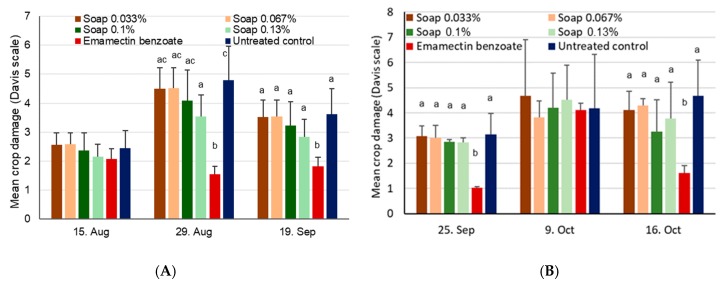
Mean crop damage induced by FAW + SD after application of alata samina, a local soap, at different concentrations during vegetative growing stages conducted in the 2018 season. For a given date, bars sharing the same letters are not significantly different from each other, while missing letters indicate a lack of differences. (**A**) Site 1 near Wa, Upper West, Ghana, where treatments were applied on 16 August, 30 August, and 13 September. (**B**) Site 2 near Accra, Ghana, where treatments were applied on 20 September and 10 October.

Crop damage was not significantly different at site 1 at first (χ^2^_5,18_ = 5.3, *p* = 0.39), but this changed during the second assessment date (χ^2^_5,18_ = 56.1, *p* < 0.001) due to low numbers for the positive control (Figure 11). For this date only, significantly lower damage was found for the high concentration soap treatment compared to the untreated control (*p* < 0.05). Significant differences were also found during the third and last assessments after all three application dates (χ^2^_5,18_ = 30.2, *p* < 0.001), again solely due to low figures for the positive control.

At site 2, crop damage was again virtually absent during the first assessment date, but was significantly different on 25 September after treatments had been applied (χ^2^_5,18_ = 87.8, *p* < 0.001) and again on 16 October (χ^2^_5,18_ = 30.3, *p* < 0.001), which in both cases was driven by the low damage observed in the positive control.

**Figure 12:**
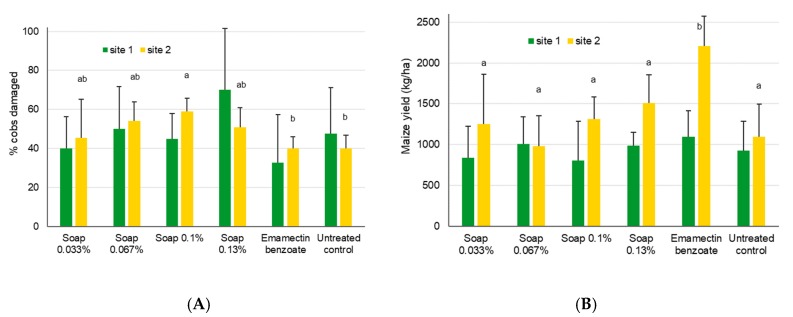
Mean maize cob damage induced by FAW + SD (**A**) and mean maize yields (**B**) after treatment with alata samina, a local soap, for site 1 near Wa, Upper West, Ghana, and site 2 near Accra, Ghana. Bars sharing the same letters are not significantly different from each other, while missing letters indicate that no significant differences were found.

There was no difference in cob damage (χ^2^_5,18_ = 8.3, *p* = 0.14) or grain yield (χ^2^_5,18_ = 2.5, *p* = 0.78) at site 1 (Figure 12). At site 2, differences were found for the cob damage (χ^2^_5,18_ = 13.5, *p* = 0.019), basically because of the 0.1% soap treatment showing higher values than both the untreated control and the insecticide treatment. Yield at site 2 also differed significantly (χ^2^_5,17_ = 30.0, *p* < 0.001), with emamectin benzoate showing higher yields compared to all other treatments. The soap treatments did not lead to any yield increases at site 1, and therefore also did not lead to positive cost/benefit values (Table 4). However, at this site, the positive control was also not effective. A significant increase in yield was found at site 2 (except for the positive control), but the cost/benefit value for the high concentration soap treatment appeared to be rather positive.

## 4. Discussion

The recent fall armyworm invasion in Africa and Asia is undoubtedly a major event in terms of pest management and food security in these continents. A number of publications have recently assessed the spread and impact of FAW, as well as farmer practices, in order to fight against the pest [7,28]. It is generally accepted that yield losses are massive, even though the few studies assessing yield losses from fields directly (e.g., Baudron et al. [4]) have indicated less severe losses as compared to studies based on farmers’ self-perception. There is a lack of studies, however, on innovative and sustainable management approaches for FAW, including local methods used by farmers in many parts of the world, despite wide agreement that there is urgent need for these [14]. We report on field trial testing of several alternative approaches for the control of FAW, aiming to help close the aforementioned knowledge gap and in light of the severe effects of FAW on maize cropping systems in Africa and Asia. Altogether, the obtained data showed high variation, as is often the case with field trials, which did not allow for small treatment effects to be separated. However, we found that the two tested neem products were as good or nearly as good as the positive control in terms of yield achieved and the cost–benefit ratio. As also shown in other studies, larval numbers were not affected as much, confirming that azadirachtin, the active ingredient in neem, mostly works as an antifeeding agent [22,29]. This is good news for small holder farmers in Africa because neem mixtures can also be home-made from neem pods or leaves [22], and have been shown to be effective against *S. frugiperda* in Zambia (unpublished data by Babendreier et al.).

In contrast to the neem-based products, published information on the pesticidal effect of maltodextrin and its use is scarce. However, a recent survey indicated that 4.5% of farmers in Ghana use it against FAW [30]. Even more Ghanaian farmers (30.6%) apply Bt against FAW, which is because of the free distribution of Bt-based biopesticides by the government. No maltodextrin (and very few biopesticides) were applied by farmers in Rwanda, Uganda, Zambia, or Zimbabwe [30]. In the present study, we found that maltodextrin was effective for one of our two test sites, with at least the highest dose nearly reaching the yields of the positive control. The cost/benefit analysis, however, indicated that it is inferior to the emamectin benzoate, at least when ignoring any potential secondary effects, such as farmer’s health or reduced impact on natural enemies. Unfortunately, few conclusions can be drawn for maltodextrin at the second site, where even the positive control did not successfully control the pest. It might be argued that pest control would be better if more than the 2 or 3 applications were done, due to the fact that this sugar-based product does not have a long activity in the field. However, costs are comparatively high for maltodextrin, thus any additional treatment would decrease the likelihood of farmers maintaining a low cost–benefit value.

For the ash- and soil-based treatments, reports indicate that a number of farmers use these methods in Africa [6,7,31], as well as in the Americas [25]. In the present paper, however, even though larval numbers appeared to be lower, no evidence was found that plant damage can be mitigated or that the yield can be increased by any of the applied treatments. A recent study showed that less than 1% of farmers in Ghana and about 5% of farmers in Zambia use ash for FAW control [7]. A recent paper that looked more closely into the decision making of farmers in five African countries found that between 2.5 and 17.7% of farmers apply ash or sand to the whorl, of which 48%–77% responded positively that they are efficacious, compared to 92%–97% responding positively for synthetic insecticides [30]. These studies do not indicate that applying ash, sand, or soil to the whorl has convincing efficacy against FAW, which is in line with the findings of the present paper. There is still a need for more detailed studies to better understand how this local method can potentially work, the amounts that are needed, and the best type of soil to use. It may be hypothesized that more sandy soil or even material used for constructing unpaved roads would provide stronger effects than just taking soil from farmers’ fields. More could be learnt from Central America, where soil has been used against *S. frugiperda* for a long time [25].

The soap tested here (i.e., the locally used alata samina) did not show itself to be effective either, as larval numbers, plant damage, and maize yields were basically unchanged compared with the untreated control. We are not aware of any published study testing alata samina against FAW, but it is reported to have certain fungicidal and insecticidal effects. For instance, Forchibe et al. [32] found that alata samina at a higher w/v concentration of 2.5% is effective to some extent for managing aphids on cabbage in Ghana.

Looking at the four trials reported on here and our two trial sites, the insecticide emamectin benzoate showed very good performance in six out of the eight cases. In two situations, yield more than doubled as compared to the negative control and the cost–benefit values were highest throughout the trials, with only one of the neem products reaching similar levels. This provides evidence for good activity for at least this tested product and helps explain why most farmers in the recently invaded area use pesticides. Emamectin benzoate is not known to cause human health hazards, however, many African smallholder farmers use other, often cheap chemical pesticides, such as lambda-cyhalothrin and cypermethrin, or even more hazardous ones, such as monocrotophos, dichlorvos, and methomyl [6], for which pest control efficacy might be lower and detrimental effects on human health are expected [18].

It is now more accepted that farmers should not panic if FAW infests their maize. They should, however, closely monitor the field situation to see whether rain events or natural enemies reduce the population levels below the economic threshold level. When action to control FAW would finally be needed, however, is still a rather difficult decision to make [15]. There is unfortunately substantial uncertainty regarding the relationship between FAW infestation levels, crop damage, and yield loss, as well as regarding the effectiveness of chemical pesticides, especially when applied by smallholder farmers without proper equipment or training. In addition, the calculation of economic thresholds generally ignores many costs, especially for toxic, broad-spectrum insecticides, including damage to the pest control services provided by natural enemies and risks to human health and the environment. A specific issue arises for substances such as neem, which, as also shown here, acts mostly as an antifeedant, making it difficult to make decisions for treatments based on larval numbers. For the experimental design here, we used fixed intervals between applications. It can be hypothesized that treatments based on careful monitoring might have led to even better results, in particular for the cost–benefit ratio.

In addition to careful monitoring of the maize crop, it is also strongly advised to ensure a generally healthy crop by considering soil health, fertilization, and even an appropriate number of plants per m^2^, which will help in reducing damage and yield loss, even in the case of severe FAW attack. It is also widely acknowledged that a number of cultural control methods would be important to reduce FAW incidence and the severity of attack [13].

In the present study, we not only assessed the efficacy of the tested products but also calculated a cost–benefit ratio, which is an indicator of the relative economic performance of the treatments. A ratio of more than 1 indicates the economic viability of the treatment compared with the control treatment. As in some treatments even less maize was harvested than in the untreated control, obviously negative cost–benefit ratios were obtained for those, which for this reason they cannot be recommended to farmers based on the present data. Altogether, the cost–benefit ratios obtained here ranged from the abovementioned slightly negative values up to a maximum value of 11.3, thus a significant return on investment in plant protection is possible. Other studies have found ratios in the same range or even higher, particularly when cash crops are considered (e.g., Amoabeng et al. [33], who worked on various botanicals in cabbage). Nevertheless, the data presented here indicate that smallholder maize farmers can save a lot of money by choosing an effective plant protection product against FAW.

Here, we only tested a small number of potential products and local methods, of which unfortunately several were shown to not be effective. Nevertheless, we believe that more research would be needed, for example for soil, sand, or soaps. There could be different types of materials, different concentrations, or different ways of applying the product that could potentially have a better effect. Also, interaction of these methods with other factors, of which rain is likely an important one, should be further elucidated. Lastly, there are hundreds of plant species with pesticidal properties that have been reported from Africa through farmer surveys and follow-up research, many of which have been confirmed to be active against a range of arthropod pests [34]. Thus, there is potential to find even more biopesticides that are effective against FAW, which would allow farmers to move away from broad-spectrum chemical insecticides. However, support from the government in one way or another (as done in Ghana) would be important to overcome issues related to product costs, and more training for farmers would also be highly relevant for increasing uptake.

## 5. Conclusions

In response to the need to achieve sustainable control of FAW, field trials were conducted on maltodextrin, neem-based products, ash, and soil, as well as the locally produced alata samina soap in Ghana. The insecticide emamectin benzoate, which was considered the positive control in this set of trials, overall showed good results in terms of damage reduction, yield increase, and cost–benefit values. If a chemical insecticide were to be chosen, emamectin benzoate might be more preferable than some of the “older” broad-spectrum ones. Nearly as efficient were the neem-based products Ozoneem and Grow-Safe. Neem-based products can, therefore, be recommended as an important component for any integrated pest management scheme to fight FAW. The high dose treatments of the two neem-based products did not have any better effects than the lower dosages, so the reduced dose may be recommended, which also has benefits in terms of optimizing the cost–benefit ratio. The starch-based maltodextrin was only efficient at one of the two test sites, with a clear dose-dependent effect. The relatively high product costs, however, resulted in overall reduced cost–benefit values. Ash and soil applied to the whorl, as well as alata samina soap treatments, did not efficiently reduce FAW larval numbers or crop damage, and thus did not significantly increase maize yields. We still propose further studies with these latter substances, for example with different types of soil, different application methods, or combining them with other insecticidal substances. Since they have very low costs associated with them, any positive effect would immediately increase the farmers’ income.

## Figures and Tables

**Table 1:** Net profits and cost–benefit values for the effect of maltodextrin-based treatments (Ma) aiming to control fall armyworm in maize in Ghana compared to the negative control. Emamectin benzoate (EB) was applied as a positive control.

Treatments	Site 1 (Wa, Upper West)		Site 2 (Near Accra)	
	Net Profit (GH¢)	Cost/Benefit Ratio	Net Profit (GH¢)	Cost/Benefit Ratio
Ma 0.27%	419.0	1.52	−764.7	−3.75
Ma 0.53%	753.8	1.74	348.1	1.13
Ma 0.8%	1136.9	1.93	−586.4	−1.42
EB 0.167%	2035.8	11.31	692.8	4.95

**Table 2:** Net profits and cost–benefit values for the effects of neem-based treatments aiming to control fall armyworm in maize in Ghana compared to the negative control. Emamectin benzoate (EB) is applied as a positive control.

Treatments	Site 1 (Wa, Upper West)		Site 2 (Near Accra)	
	Net Profit (GH¢)	Cost–Benefit	Net Profit (GH¢)	Cost–Benefit
Ozoneem 0.33%	821.7	2.11	−104.2	−0.37
Ozoneem 0.17%	1615	6.33	441.1	2.32
Grow-Safe 0.33%	1965.1	8.45	8.4	0.05
Grow-Safe 0.17%	1876.3	10.65	265.2	1.92
EB 0.17%	1885.0	10.47	743.4	5.31

**Table 3:** Net profit and cost–benefit values for the effects of ash- and soil-based treatments aiming to control fall armyworm in maize in Ghana compared to the negative control. Emamectin benzoate (EB) was applied as a positive control.

Treatments	Site 1 (Wa, Upper West)		Site 2 (Near Accra)	
	Net Profit (GH¢)	Cost–Benefit Ratio	Net Profit (GH¢)	Cost–Benefit Ratio
Ash powder	-5.06	−0.04	282.62	2.83
Ash solution	54.2	0.45	102.94	1.03
Local soil	−67.5	−0.56	−119.94	−1.20
Ash and soil	37.5	0.31	253.36	2.53
EB 0.167%	982.4	5.46	182.18	1.14

**Table 4:** Net profit and cost–benefit values for the effect of alata samina soap treatments (AS) at four concentrations aiming to control fall armyworm in maize in Ghana compared to the negative control. Emamectin benzoate (EB) was applied as a positive control.

Treatments	Site 1 (Wa, Upper West)		Site 2 (Near Accra)	
	Net Profit (GH¢)	Cost–Benefit Ratio	Net Profit (GH¢)	Cost–Benefit Ratio
AS 0.033%	−244.3	−2.02	218.5	2.17
AS 0.067%	−13.8	−0.11	−324.6	−3.20
AS 0.1%	−297.2	−2.42	342.8	3.36
AS 0.13%	−46.6	−0.38	723.0	7.04
EB 0.17%	48.3	0.27	2087.8	14.91
